# PKM2 enhances chemosensitivity to cisplatin through interaction with the mTOR pathway in cervical cancer

**DOI:** 10.1038/srep30788

**Published:** 2016-08-05

**Authors:** Haiyan Zhu, Jun Wu, Wenwen Zhang, Hui Luo, Zhaojun Shen, Huihui Cheng, Xueqiong Zhu

**Affiliations:** 1 Department of Obstetrics and Gynecology, the Second Affiliated Hospital of Wenzhou Medical University, Wenzhou 325027, China

## Abstract

Pyruvate kinase M2 (PKM2) is a key driver of aerobic glycolysis in cancer cells and has been shown to be up-regulated by mTOR *in vitro*. Our previous proteomic profiling studies showed that PKM2 was significantly upregulated in cervical cancer tissues after treatment with neoadjuvant chemotherapy (NACT). Whether PKM2 expression predicts cisplatin-based NACT sensitivity and is mTOR dependent in cervical cancer patients remains unclear. Using paired tumor samples (pre- and post-chemotherapy) from 36 cervical cancer patients, we examined mTOR, HIF-1α, c-Myc, and PKM2 expression in cervical cancer samples and investigated the response to cisplatin-based NACT. In addition, we established PKM2 suppressed cervical cancer cell lines and evaluated their sensitivity to cisplatin *in vitro*. We found that the mTOR/HIF-1α/c-Myc/PKM2 signaling pathway was significantly downregulated in post-chemotherapy cervical cancer tissues. High levels of mTOR, HIF-1α, c-Myc, and PKM2 were associated with a positive chemotherapy response in cervical cancer patients treated with cisplatin-based NACT. *In vitro*, PKM2 knockdown desensitized cervical cancer cells to cisplatin. Moreover, PKM2 had complex interactions with mTOR pathways. mTOR, HIF1α, c-Myc, and PKM2 expression in cervical cancer may serve as predictive biomarkers to cisplatin-based chemotherapy. PKM2 enhances chemosensitivity to cisplatin through interaction with the mTOR pathway in cervical cancer.

Although several advances in screening, vaccination, diagnostic and treatment modalities have been made, cervical cancer remains a leading cause of cancer-related death in women, accounting for nearly 3.7% of the total newly-diagnosed cancer cases and 3.2% of total cancer deaths^1^. Specifically, patients with bulky (>4 cm in diameter) stage IB-IIA cervical cancers exhibit a poorer prognosis compared to those with a tumor ≤4 cm, regardless of treatment selection^2^. Since the mid-1980s, neoadjuvant chemotherapy (NACT) prior to surgery has been widely used to treat bulky cervical cancer, which significantly improved patient prognosis and reduced the risk factors for recurrence^3^. However, not all patients respond equally to chemotherapy and those who have a poor response to NACT tend to develop resistance to radiotherapy and have poor therapeutic outcomes^3^. Moreover, cancer patients receiving chemotherapeutic treatments routinely experience a wide range of distressing side effects, including anemia, neutropenia, nausea, and neurotoxicity^4^. Therefore, effective and, predictive protein biomarkers that can differentiate patients into responders and non-responders would greatly optimize chemotherapy outcomes for cancer patients.

Previously, by using proteomics profiling to investigate paired cervical cancer samples taken pre- and post-chemotherapy from 6 NACT-sensitive patients, we identified 116 proteins that were significantly changed^5^. In all, 31 proteins were analyzed by MALDI-TOF/TOF MS, and 16 proteins were downregulated in the cancer tissue after NACT relative to the level before chemotherapy, including pyruvate kinase M2 (PKM2)^5^. PKM2 is an important cancer metabolism enzyme responsible for the Warburg effect. In highly glycolytic cancers, the conversion of pyruvate to lactic acid and ATP in the presence of oxygen (‘aerobic glycolysis’) generates the necessary amount of energy needed for rapid cellular proliferation^6^. Indeed, enhanced expression of PKM2 is frequently observed in various human cancers and is important for tumor growth^7^. However, accumulating evidence has suggested that PKM2 is more than just a regulator of metabolic reprogramming, but it is also a potential biomarker for chemotherapy response.

Recent research has indicated that PKM2 upregulation promotes chemosensitivity in breast cancer, gastric cancer and intestinal cancer^8^,^9^,^10^. High expression of PKM2 was associated with chemosensitivity to epirubicin and 5-fluorouracil in breast cancer^8^, whereas decreased PKM2 activity was linked to platinum-based drug resistance in human gastric and colorectal cancer^9^,^10^. However, whether PKM2 expression correlates with cisplatin-based NACT chemosensitvity in cervical cancer patients is currently unknown.

mTOR (mammalian target of rapamycin), also known as FRAP1 (FKBP 12-rapamycinassociated protein 1), is an important cell growth serine-threonine kinase that is frequently dysregulated in human cancers^11^. Recently, Sun *et al*.^12^ reported that mTOR-induced PKM2 expression in mouse kidney tumors was through hypoxia-inducible factor-1α (HIF-1α), which was previously shown by Land *et al*. to interact with regulatory associated protein of mTOR^13^. In addition, the former group demonstrated that mTOR could up-regulate the transcription factor c-Myc, which then turns on the splicing factors necessary for PKM2 expression. Whether mTOR, HIF-1α, and c-Myc are involved with PKM2 expression in cervical cancer patients receiving cisplatin-based NACT remains unclear.

In this study, we examined the correlation between mTOR, HIF-1α, c-Myc, and PKM2 expression and the response to cisplatin-based NACT in patients with stage IB2 or IIA2 cervical cancer. Moreover, we established PKM2 suppressed cervical cancer cell lines and evaluated their sensitivity to cisplatin and interaction with mTOR pathway.

## Results

### Patient characteristics

A total of 36 patients were enrolled in this study. Among the 36 patients, complete response (CR) and partial response (PR) was achieved in 7 and 12 patients, respectively, while stable disease (SD) and progressive disease (PD) was observed in 11 and 6 patients, respectively. The patients with CR or PR were defined as chemotherapy responders, while the patients with SD or PD were deemed as chemotherapy non-responders. Thus, the overall response rate to NACT was 52.7%. No significant differences were noted in age at diagnosis, the disease’s FIGO stage, tumor size, and pathological grade between the chemotherapy responder and non-responders (*P* > 0.05) (Table 1).

### Proteins expression in cervical cancers pre- and post- chemotherapy

**Figure 1:**
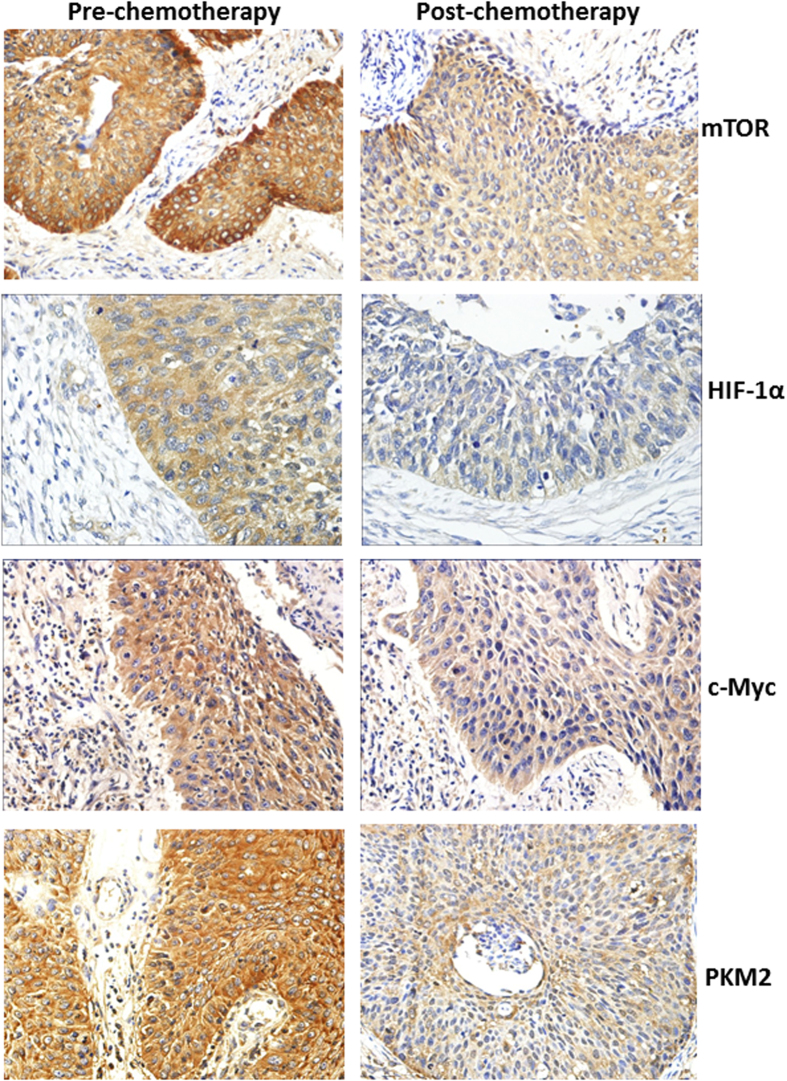
The expression of mTOR, HIF-1α, c-Myc, and PKM2 in paired samples of cervical cancer as compared to corresponding pre- chemotherapy and post- chemotherapy (SP staining, ×400). mTOR, HIF-1α, c-Myc, and PKM2 protein expression in cervical cancer tissues were detected mainly in the cytoplasm. Pre-chemotherapy cervical cancer tissues consistently showed moderate or intense positive staining of mTOR, HIF-1α, c-Myc, and PKM2, while post-chemotherapy tissue consistently showed weak or moderate positive staining.

**Figure 2:**
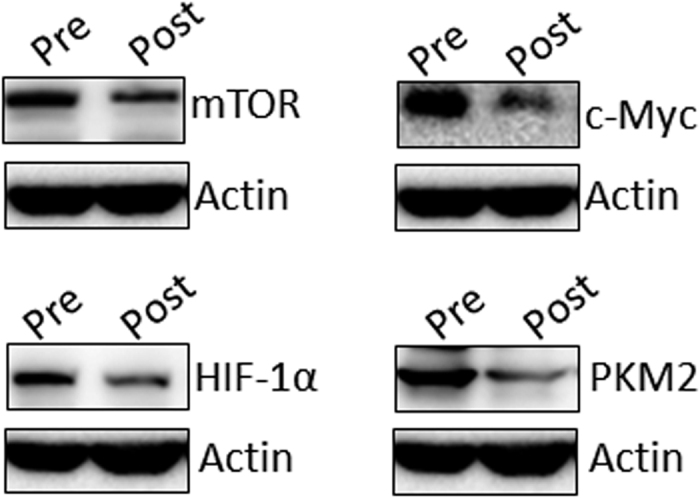
The expression of mTOR, HIF-1α, c-Myc, and PKM2 proteins in matched pairs of pre- and post-chemotherapy primary cervical cancer biopsies by Western blot analysis. mTOR, HIF-1α, c-Myc, and PKM2 protein expression were markedly downregulated in post-chemotherapy tissues compared to their matched pre-chemotherapy tissues.

In the present study, we first evaluated the effect of chemotherapy on the expression of mTOR, HIF-1α, c-Myc, and PKM2 in cervical cancer samples. Proteins extracted from 36 matched primary cervical cancer biopsies before and after cisplatin-based NACT were quantitated using immunohistochemistry. As shown in Fig. 1, mTOR, HIF-1α, c-Myc, and PKM2 protein expression in the tumor tissues were detected mainly in the cytoplasm. Pre-chemotherapy cervical cancer tissues consistently showed moderate or intense positive staining of mTOR, HIF-1α, c-Myc, and PKM2, while post-chemotherapy tissue consistently showed weak or moderate positive staining. Using a Wilcoxon test, mTOR, HIF-1α, c-Myc, and PKM2 expression were significantly decreased in post-chemotherapy samples compared with pre-chemotherapy tissue samples, respectively (*P* < 0.05) (Table 2). To confirm these results, we further analyzed the tumor expression of mTOR, HIF-1α, c-Myc, and PKM2 in matched pairs of primary cervical cancer biopsies by western blotting analysis. As expected, mTOR, HIF-1α, c-Myc, and PKM2 protein expression were markedly downregulated after cisplatin-based NACT when compared to their matched pre-chemotherapy tissues, consistent with our previous results (Fig. 2).

### Proteins expression in cervical cancers between chemotherapy-response and non-response group

We then investigated the involvement of mTOR, HIF-1α, c-Myc, and PKM2 proteins in predicting cisplatin chemosensitivity. We examined the tumor expression of mTOR, HIF-1α, c-Myc, and PKM2 in pre-chemotherapy cervical cancer tissues between chemotherapy responders and non-responders by immunohistochemistry. Patients with high mTOR, HIF-1α, c-Myc, and PKM2 levels were more sensitive to cisplatin-based NACT than those with low protein expression (*P* < 0.05, respectively) (Table 3).

### Influence of cisplatin on PKM2 and mTOR expression *in vitro*

**Figure 3:**
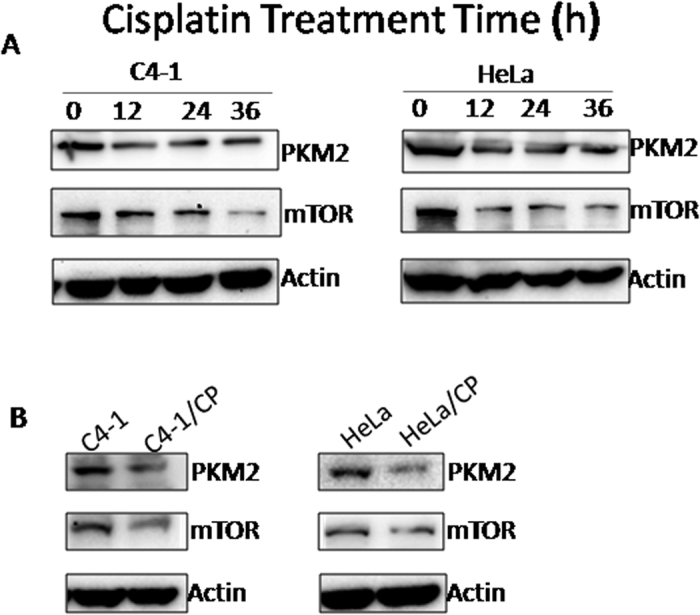
Effect of cisplatin on the expression of PKM2 and mTOR *in vitro*. (**A**) C4-1 and HeLa cells were treated with 10 μM cisplatin for 12 h, 24 h and 36 h, respectively, and then followed by western blot analysis. (**B**) Western blot analysis of PKM2 and mTOR expression in C4-1 and HeLa cells and their cisplatin-resistant sublines (C4-1/CP and HeLa/CP).

In the next series of studies, we investigated the relationship between PKM2 expression and cisplatin sensitivity using two cervical cancer cell lines, C4-1 and HeLa. In a first set of experiment, we treated C4-1 and HeLa cells with 10 μM of cisplatin for 12 h, 24 h or 36 h, and then followed by western blotting analysis. In both C4-1 and HeLa cells, cisplatin significantly reduced PKM2, and mTOR expression at all studied time points (12, 24 and 36 h) (Fig. 3A). Furthermore, the expression of PKM2 and mTOR was significantly decreased in cisplatin-resistant HeLa and C4-1 cells compared to parental cell lines (Fig. 3B).

### Influence of PKM2 knockdown on chemosensitivity

**Figure 4:**
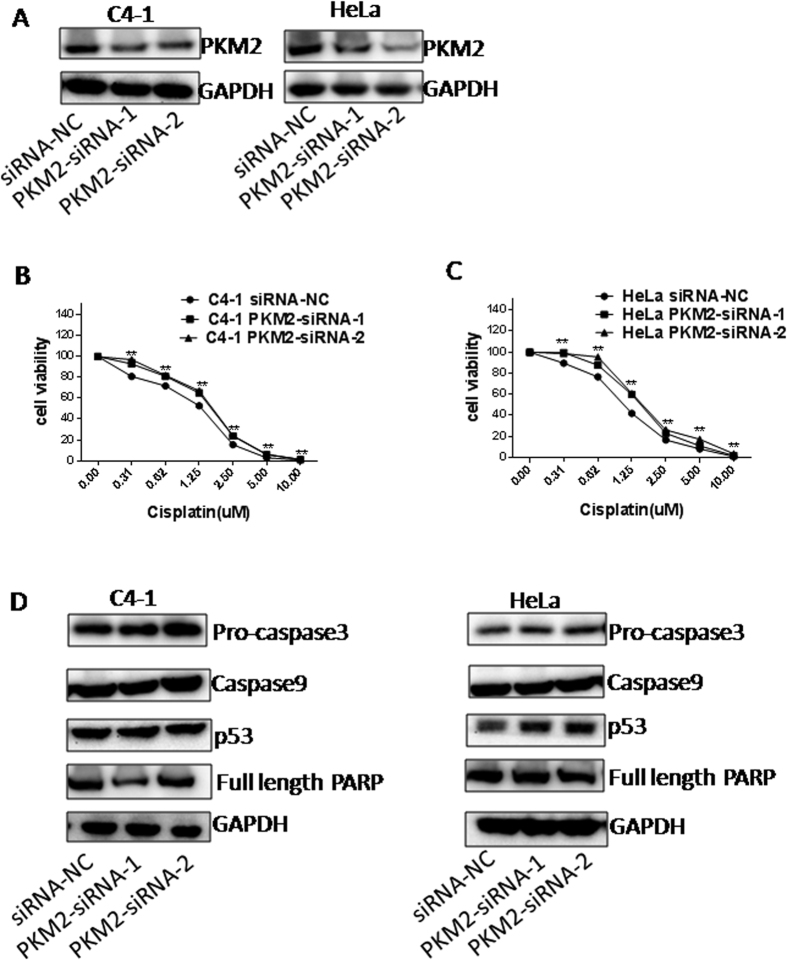
Influence of PKM2 knockdown on chemosensitivity to cisplatin in C4-1 and HeLa cells. (**A**) Confirmation of PKM2 silencing in C4-1 and HeLa cells by Western blotting. (**B,C**) Cells were treated with different concentrations of cisplatin (0, 0.31, 0.62, 1.25, 2.5, 5, 10 μM) for 5 days. Cell viability was analyzed by MTT assay for chemosensitivity. The chemosensitivity of C4-1 and HeLa cells to cisplatin was significantly decreased by transient transfection with PKM2- siRNA-1 and PKM2- siRNA-2 compared with negative control (NC). *P < 0.05; **P < 0.01. (**D**) Western blot analysis of pro-caspase3, caspase9, full length-PARP and p53 expression in siRNA-NC, PKM2- siRNA-1 and PKM2- siRNA-2- C4-1 cells as well as HeLa cells.

To further investigate the effect of PKM2 on cisplatin sensitivity in cervical cancer cells, we knocked down PKM2 using two small-interfering RNAs (PKM2-siRNA-1 and PKM2-siRNA-2) in C4-1 and HeLa cell lines. Expectedly, as shown in Fig. 4, transfection of cells with PKM2 siRNAs significantly suppressed PKM2 expression, which was confirmed by western blotting (Fig. 4A). Silencing PKM2 increased the cell viability of both C4-1 and HeLa cells following treatment with various concentrations of cisplatin for 48 h (Supplementary Figure 1A), 5 days (Fig. 4B,C) or 7 days (Supplementary Figure 1B) as determined by MTT. The IC_50_ value to cisplatin for C4-1/siRNA- NC cells, C4-1/PKM2- siRNA-1 cells and C4-1/PKM2- siRNA- 2 cells was 2.32 ± 0.40, 3.26 ± 0.49, 3.14 ± 0.47 μM for 48 h; 1.04 ± 0.34, 1.42 ± 0.16, 1.51 ± 1.16 μM for 5 days and 0.77 ± 0.25, 1.26 ± 0.15, 1.09 ± 0.14 μM for 7 days (Supplementary Figure 1C), suggesting that higher cisplatin IC_50_ correlated with PKM2 suppression. Similarly, the IC_50_ value to cisplatin for HeLa/PKM2- siRNA-1 cells and HeLa/PKM2- siRNA- 2 cells was significant increased compared with HeLa/siRNA- NC cells for 48 h, 5 days or 7 days (Supplementary Figure 1D). These results supported the notion that loss of PKM2 rendered the cells more resistant to cisplatin. Of note, there was no change in apoptosis associated proteins such as pro-caspase 3, caspase 9, full length-PARP and p53 expression following PKM2 knockdown (Fig. 4D).

### The mTOR pathway was involved in PKM2 modulated cisplatin sensitivity in cervical cancer

**Figure 5:**
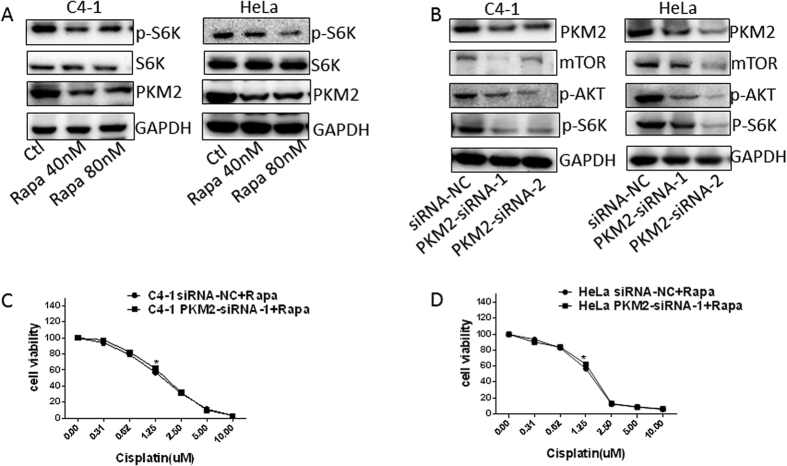
The mTOR pathway was involved in PKM2 modulated cisplatin sensitivity in cervical cancers. (**A**) After treatment with 40 nM and 80 nM rapamycin for 24 h, expression of p-S6K, S6K and PKM2 in C4-1 and HeLa cells was determined by western blot analysis. (**B**) Western blot analysis of PKM2, mTOR, p-S6K and p-AKT expression in PKM2- siRNA C4-1 cells and HeLa cells. (**C,D**) Cells were treated with or without 40 nM rapamycin and different concentrations of cisplatin (0, 0.31, 0.62, 1.25, 2.5, 5, 10 μM) for 4 days. Cell viability was analyzed by MTT assay for chemosensitivity.

Recent studies have identified PKM2 as a target gene of mTOR. Silencing mTOR with siRNA decreased PKM2 protein levels in various cancer cell lines. Therefore, we postulated that mTOR pathway was involved in PKM2 modulated cisplatin sensitivity in cervical cancers. To test this assumption, the mTOR inhibitor rapamycin (40 nM or 80 nM) was used to treat C4-1 and HeLa cells for 24 h. Upon rapamycin treatment, we observed inhibition of mTOR signaling in both cervical cancer cell lines as evident from reduced phosphorylation of S6K upon rapamycin treatment (Fig. 5A). Inhibition of mTOR with rapamycin also decreased PKM2 expression in these two cell lines (Fig. 5A). Interestingly, silencing PKM2 significantly reduced the protein levels of mTOR, p-S6K and p-AKT in C4-1 and HeLa cells (Fig. 5B). These results indicated an interaction between mTOR pathway and PKM2 in cervical cancer. To evaluate whether this interaction involved in modulating cisplatin sensitivity in cervical cancer, we then investigated the effect of suppression of PKM2 on cisplatin sensitivity with additional mTOR inhibition. Interestingly, inhibiting mTOR pathway by rapamycin blocked silencing-PKM2-induced chemoresistance both in C4-1 cells (Fig. 5C) and HeLa cells (Fig. 5D). Additionally, combination treatment with cisplatin and rapamycin resulted in an additive effect on suppressing PKM2 expression (Supplementary Figure 2A). Cisplatin treatment and PKM2 silencing together resulted in an additive effect on inhibiting mTOR pathway (Supplementary Figure 2B). Altogether, these results revealed that the mTOR pathway may interact with PKM2 to modulate cisplatin sensitivity in cervical cancer.

## Discussion

In this study, we demonstrated that mTOR, HIF-1α, c-Myc and PKM2 expression were significantly downregulated in post-chemotherapy cervical cancer tissues compared with matched pairs of pre-chemotherapy biopsies. High levels of mTOR, HIF-1α, c-Myc, and PKM2 expression were associated with a positive cisplatin-based NACT response. These findings suggest that mTOR, HIF-1α, c-Myc, and PKM2 may serve as potential biomarkers to predict cisplatin chemosensitivity. Furthermore, we found that knockdown of PKM2 desensitized cervical cancer cells to cisplatin, supporting the notion that PKM2 promotes sensitivity to cisplatin in cervical cancer.

The molecular mechanisms underlying cisplatin resistance are complex and may include: reduced import and increased efflux of platinum compounds, increased DNA damage repair responses, inactivation of apoptosis, transcription factors, activation of epithelial-to-mesenchymal transition (EMT), and changes in DNA methylation, microRNA expression and cancer stem cell characteristics^14^. Intriguingly, our study revealed that PKM2 knockdown had no effect on apoptosis protein levels such as caspase 3, caspase 9, PARP and p53 in HeLa and C4-1 cell lines. In the past few years many studies have directed their attention to cancer cell metabolism as a mechanism by which cancer cells become resistant to chemotherapy. Indeed, cancer cells are extremely cunning in their ability to rewire their metabolism and regulate chemosenstivity, thereby affecting the chemotherapeutics response^15^.

Surprisingly, we observed low expression of the glycolytic enzyme PKM2 in PKM2-expressing cervical cancer patient tissues following chemotherapy, suggesting cervical cancer cells adapt to cisplatin’s toxicity by relying less on aerobic glycolysis pathways. Furthermore, PKM2 expression was found to be an independent negative prognostic factor in cervical cancer patients^16^. Recently, the association between PKM2 expression and chemoresistance was investigated in several types of cancer albeit with conflicting results. Decreased PKM2 protein expression and activity was found in cisplatin-resistant gastric cancer cells^9^, oxaliplatin-resistant colorectal cell lines^10^, and cisplatin-resistant ovarian cancer cell lines^17^. In agreement with the above studies, high expression of PKM2 was associated with a higher response rate in oxaliplatin-treated colorectal cancer patients^10^ and chemosensitivity to epirubicin and 5-fluorouracil in breast cancer^8^. In contrast, strong PKM2 expression was associated with poor response to chemotherapy in esophageal squamous cell carcinoma patients^18^. PKM2 inhibition decreased non-small cell lung cancer chemoresistance to anticancer drugs, including docetaxel and cisplatin^19^,^20^. In this study, we found that patients with high PKM2 expression were more sensitive to cisplatin-based NACT than those with low protein expression, which was confirmed *in vitro*. The expression of PKM2 was attenuated in cisplatin-resistant HeLa and C4-1 cells compared with their parental cell lines. Consistent with these findings, our data shows that suppression of PKM2 decreased cisplatin sensitivity in C4-1 and HeLa cells. Thus, loss of PKM2 confers a selective, survival advantage in cervical cancer cells treated with cisplatin. One possible benefit for depleting PKM2 is that it forces cancer cells to shift their energetic currency from glucose to glutamine or fatty acids. This metabolic alteration could maintain energy production for the cancer cell and thereby promote ATP-dependent signaling processes that mediate cisplatin resistance^21^,^22^. Nevertheless, the molecular mechanism of how PKM2 regulates chemosensitivity remains unclear. Taken together, our results strongly suggest that PKM2 expression is associated with cisplatin sensitivity and expression of pre-chemotherapy PKM2 may be used as a predictive biomarker for platinum-based NACT efficacy in cervical cancer patients.

Another possibility by which low PKM2 confers chemoresistance is through the mTOR signaling pathway. A recent study showed that PKM2 expression is induced by an mTOR/HIF-1α/c-Myc glycolysis signaling network, promoting aerobic glycolysis in tumor cells^12^. In cervical cancer, mTOR protein expression was significantly increased in cancer tissues compared to normal cervical tissue^23^,^24^. Similarly to previous reports, our study indicated that mTOR may serve as a predictive biomarker to chemotherapy response in cervical cancer patients^23^,^24^,^25^. Furthermore, our results showed that high levels of mTOR were associated with chemosenstivity and increased PKM2 expression. Inhibiting mTOR signaling by rapamycin notably downregulated PKM2 expression, while PKM2 suppression by siRNAs greatly reduced mTOR, p-S6K and p-AKT, suggesting PKM2 can interact with mTOR signaling. Furthermore, inhibiting mTOR pathway by rapamycin blocked silencing-PKM2-induced chemoresistance both in C4-1 cells and HeLa cells, suggesting that PKM2 modulating cisplatin sensitivity partially controlled through mTOR signaling.

The current study also demonstrated that cisplatin decreased c-Myc and HIF-1α expression and strong c-Myc and HIF-1α expression was significantly correlated with a positive response to cisplatin-based NACT in patients with cervical cancer. These findings are in agreement with those from recently reported studies that proposed c-Myc and HIF-1α work together to regulate chemosensitivity. c-Myc is a key regulator of cell proliferation, and is hyper-activated in many cancers^26^. The amplification and overexpression of c-Myc have been detected in both cervical cancer cell lines and tissues^27^. In addition to its role in tumorigenesis, an association between c-Myc expression and drug resistance has been investigated in some types of cancers, however with conflicting results. c-Myc expression was found to be upregulated in docetaxel-resistant residual prostate cancer cell lines^28^ and cisplatin-resistant gastric cancer SGC7901 compared with their parental cells^29^. However, c-Myc amplification in breast cancer was protective and the patients were sensitive to chemotherapy in contrast to patients without amplification in metastatic breast cancer^30^,^31^. Additionally, Soh *et al*.^32^ suggested that c-Myc overexpression was an independent prognostic marker in cervical cancer. With respect to human cervical cancer, few studies have evaluated the possible role of c-Myc in chemotherapy response. Using proteomics profiling, Yim *et al*. reported c-Myc was down-regulated in HeLa cells following treatment with several chemotherapy agents such as paclitaxel, cisplatin and 5-fluorouracil^33^,^34^,^35^. In agreement with previous studies, our data also showed down-regulation of c-Myc in both cervical cancer cells and cervical cancer tissues following cisplatin treatment. Furthermore, we detected that c-Myc expression was statistically higher in the chemotherapy-response group than in the non-response group, suggesting a higher level of c-Myc to be a potential predictive biomarker for cisplatin chemosensitivity in cervical cancer.

HIF-1α is a transcription factor that immortalizes tumors by inducing key genes in cancer biology, including cell growth and glycolysis genes^36^. Thus, overexpression of HIF-1α is associated with resistance to cancer chemotherapy and increased patient mortality in several cancer phenotypes^36^,^37^. Along similar lines, previous studies showed that HIF-1α was overexpressed in cervical cancer cells both *in vitro* and *in vivo*^38^,^39^, and higher HIF-1α expression was associated with lower 5-years overall survival rate and 5-years disease free survival rate^40^. In the present study, our data provides evidence that high expression of HIF-1α was significantly correlated with a positive response to chemotherapy in cervical cancer. This finding re-confirms our notion that highly glycolytic cancer cells are extremely responsive to cisplatin.

Since mTOR, HIF-1α, c-Myc, and PKM2 play a crucial role in regulating cell metabolism, our results provide support and rationale for targeting metabolic pathways to enhance the efficacy of common therapeutic agents or overcome resistance to chemotherapy. Our study identified four cell metabolism associated proteins, mTOR, HIF-1α, c-Myc, and PKM2, as potential biomarkers to predict the response to cisplatin in cervical cancer patients. Surprisingly, PKM2 expression was down-regulated in cisplatin-resistant cells, suggesting cervical cancer cells adapt to cisplatin by rewiring their energy metabolism. Our results also suggested PKM2 enhanced sensitivity to cisplatin through interaction with the mTOR signaling pathway in cervical cancer, indicating that PKM2 may be a promising target for therapeutic approaches in cervical cancer, as well as other cancers.

## Materials and Methods

### Ethics Statement

This study was approved by the Institute’s Review Board of the Second Affiliated Hospital of Wenzhou Medical University and conducted according to the Helsinki declaration. Informed consent was obtained from all subjects prior to participation in the study.

### Patients and tissue specimens

Between January 2007 and August 2012, 36 patients with IB2 or IIA2 (bulky, primary tumor >4 cm in diameter) cervical cancer were recruited for a pilot study aimed at developing predictive biomarkers for response to chemotherapy. None of the patients had received chemotherapy, immunotherapy, hormonal therapy or radiotherapy before the specimen collection. The histological classifications and clinical staging were based on the International Federation of Gynecology and Obstetrics classification system. Median patient age was 44y (range, 25–62 y). Paired tumor samples (pre- and post-chemotherapy) from all these patients were obtained.

### Treatment schedule and evaluation of treatment

All eligible patients received one or two cycles of cisplatin-based NACT. The schedule of the NACT was as follows: cisplatin (60 mg/m^2^), 5-fluorouracil (750 mg/m^2^) and mitomycin (8 mg/m^2^) were administrated via uterine artery injection. Cycles were repeated every 4 weeks. After one to two cycles of chemotherapy, it was decided whether radical hysterectomy was possible. The chemotherapy response was evaluated by measuring the tumor’s two dimensions (the longest diameter and its perpendicular diameter) with magnetic resonance imaging or other radiographic means. A complete response (CR) was defined as the complete remission of the tumor. A partial response (PR) was defined as a 50% or more decrease in the tumor volume. Stable disease (SD) meant a steady state or a response less than 50%, and progressive disease (PD) was defined as an unequivocal increase of at least 25% in the tumor volume. The patients with CR or PR were defined as chemotherapy responders, while the patients with SD or PD were deemed as chemotherapy non-responders. All patients were treated with radial hysterectomy and bilateral pelvic lymphadenectomy two to three weeks after completion of the NACT regimen as described previously^3^.

### Cell cultures and reagents

Human cervical cancer cell lines (C4-1 and HeLa) were originally obtained from the American Type Culture Collection (ATCC). Their cisplatin-resistant sublines (C4-1/CP and HeLa/CP) were obtained by *in vitro* selection with cisplatin. The culture was maintained in RPMI 1640 medium (Invitrogen, Carlsbad, CA, USA) supplemented with 10% fetal bovine serum (FBS) and 0.1% penicillin in 5% CO2 at 37 °C. For drug treatment, cisplatin (Sigma) and rapamycin (Sigma) were dissolved in water and dimethylsulfoxide (DMSO) respectively; aliquots were stored at −80 °C. Different concentration of cisplatin (0, 0.31, 0.62, 1.25, 2.5, 5, 10, 20 μM) was used to treat cervical cancer lines for 48 h, 5days or 7days *in vitro* sensitivity assay. And, 10 μM cisplatin (12 h, 24 h and 36 h treatment) was used to investigate the impact of the cisplatin on PKM2 and mTOR expression in cervical cancer cells. As for rapamycin, 40 nm or 80 nm rapamycin (24 h treatment) were used to inhibit mTOR pathway. To evaluate the effect of suppression of PKM2 on cisplatin sensitivity with additional mTOR inhibition, SiRNA-NC and PKM2- siRNA-1-C4-1 as well as HeLa cells were treated with different concentrations of cisplatin (0, 0.31, 0.62, 1.25, 2.5, 5, 10 μM) and 40 nm rapamycin for 4 days.

### Knockdown analysis using PKM2-siRNAs

Cells were seeded at 60% confluence in 6, 12 and 96 well plates, depending on the following experiments. PKM2 was transiently silenced by using two different small-interfering RNAs (siRNA-1, -2) targeting PKM2 (Sigma). A silencer negative transcription control (Sigma) was used in each experiment. Transfection was performed using Lipofectamine RNAiMAX (Invitrogen) according to the manufacturer’s instructions. Forty-eight hours after transfection, whole-cell lysates were prepared for further analysis by western blotting and *in vitro* sensitivity assay as described below.

### Immunohistochemistry

Immunohistochemistry staining was performed on paraffin-embedded 4 μm sections and mounted on poly-L-lysine-coated slides. Briefly, after deparaffinization in xylenes and rehydration through graded ethanol solutions, antigen retrieval was performed by submerging the sections into a 10 μmol/L citrate buffer solution (pH6.0) for 10 minutes in a microwave oven. The tissue sections were then treated with 3% hydrogen peroxide in methanol to suppress the endogenous peroxidase activity. Tissue sections were then incubated with anti-PKM2 (CST, USA; 1:100), anti-mTOR (Abcam, Cambridge, USA; 1:100), anti-c-myc (Santa Cruz, USA; 1:200) and anti-HIF-1α (Abcam, Cambridge, USA; 1:200) for 2 h at room temperature. After washing, the sections were incubated with pre-diluted secondary antibody (Santa Cruz, USA), followed by further incubation with 3,3-diaminobenzidine tetrahydrochloride (DAB). Finally, the slides were counterstained with hematoxylin and mounted in an aqueous mounting medium. Appropriate positive and negative controls were stained in parallel. For negative controls, primary antibodies were replaced with PBS. Human lung cancer tissues were used as a positive control for PKM2, human prostate cancer tissues were used as a positive control for mTOR and human cervical cancer tissues were used as a positive control for c-myc and HIF-1α.

### Evaluation of immunoreactivity

mTOR, HIF1α, c-Myc, and PKM2 immunoreactivity were observed mainly in cytoplasm. Staining evaluation was performed by two independent observers at the department of pathology, who were blinded to the clinical outcome. Expression of the four markers was analyzed by an individual labeling score considering percent and staining intensity of positive cells^41^. Intensity of stained cells was graded semi-quantitatively into four levels: 0 (no staining); 1 point (weak staining =  light yellow); 2 points (moderate staining =  yellow brown) and 3 points (strong staining = brown); and the percentage was scored as: 0 (0 to 5%), 1 point (6% to 24%), 2 points (25% to 49%), 3 points (50% to 74%), and 4 points (75% to 100%). Intensity and fraction of positive cell scores were multiplied for each marker and thus got the immunoreactive score (IRS).

### Western Blotting

Samples were homogenized and lysed in Laemmli buffer with a cocktail of protease inhibitors. The total protein concentrations were quantified by the BCA protein assay (Thermo Scientific, Rockford, IL). Equal amounts of total protein were resolved by SDS PAGE, transferred to a nitrocellulose membrane under constant voltage and blocked with TBST containing 5% non-fat dried milk. Primary antibodies and secondary antibodies were diluted in TBST or 3% non-fat dried milk and applied with a washing step in between. Proteins were detected using the Amersham ECL western blotting detection kit (GE Healthcare, Piscataway, NJ). Primary antibodies used including: anti-PKM2 (Sigma, USA; 1:100), anti-mTOR (Abcam, USA; 1:100), anti- caspase3 (Cell signaling, USA, 1:1000), anti- caspase9 (BD, USA, 1:500), anti- PARP (Santa Cruz, USA, 1:1000), anti-p53 (Santa Cruz, USA, 1:4000), p-AKT(Cell signaling, USA, 1:500), p-S6K(Cell signaling, USA, 1:1000), S6K(Santa Cruz, USA, 1:1000).

### *In vitro* sensitivity assay (MTT Assay)

Cells were plated in a 96-well plate at 1.5∼3 × 10^4^ cells/well in triplicates and treated with various concentrations of cisplatin after overnight incubation. After exposed to cisplatin for 48 h, 5 days or 7 days, 3–19(4,5-Dimethylthiazol--yl)-2,5- diphenyltetra- zolium bromide solution (MTT, 2 mg/ml in PBS) was added and incubated at 37 °C for 2 h in a tissue incubator. The blue colored formazan product was dissolved in DMSO and measured at 595 nm wavelength on a Biotek plate reader. The cell viability was calculated by the following formula: Cell viability (%) = (OD treatment − OD blank)/(OD control − OD blank) × 100%.

### Statistical Analysis

The Kolmogorov-Smirnov test of normality was applied. Continuous variables were presented as means ± standard deviations, while non-normally distributed variables were presented as median (P25–P75). The patient profile between the NACT responsive and non-responsive group displayed a normal distribution and was analyzed using a Student t-test (age and tumor size) or chi-square test (FIGO stage and Histological grade). Protein expression between the two groups displayed a non-normal distribution and was evaluated by non-parametric tests such as the Wilcoxon test. The software of SPSS 16.0 (SPSS Inc, IL) was used for statistical analysis. A 2-tailed *P* value of <0.05 was considered to be statistically significant.

## Additional Information

**How to cite this article**: Zhu, H. *et al*. PKM2 enhances chemosensitivity to cisplatin through interaction with the mTOR pathway in cervical cancer. *Sci. Rep.* **6**, 30788; doi: 10.1038/srep30788 (2016).

## Supplementary Material

Supplementary Information

## Figures and Tables

**Table 1:** Clinical characteristics of cervical cancer patients between NACT responders and non-responders.

	Responders (n = 19)	Nonresponders (n = 17)	*P*
Age(years)	46 ± 7.6	44 ± 10.1	NS*
FIGO stage			NS*
IB2	11	10	
IIA2	8	7	
Histological grade			NS*
G1	4	4	
G2	13	10	
G3	2	3	
Tumor size (cm)	4.11 ± 1.24	4.24 ± 1.35	NS*

*P > 0.05.

**Table 2:** The expression of mTOR, HIF-1α, c-Myc, and PKM2 in tumor cells of pre- and post- chemotherapy cervical cancer.

	Pre-chemotherapy	Post-chemotherapy	Z	*P*
mTOR	8.5(6.00–10.75)	6.0(4.00–8.00)	3.050	0.002*
HIF-1α	7.0(3.00–8.00)	4.0(2.25–5.00)	2.929	0.003*
c-Myc	6.0(4.00–8.00)	3.5(2.00–6.00)	2.796	0.005*
PKM2	7.0(4.25–10.00)	4.0(2.00–8.75)	2.117	0.034*

Data are expressed as median (P25–P75), *P < 0.05.

**Table 3:** The expression of mTOR, HIF-1α, c-Myc, and PKM2 in tumor cells of pre-chemotherapy cervical cancer between chemotherapy-response and non-response group.

	Responders	Non-responders	Z	*P*
mTOR	10.0(8.00–11.00)	6.0(4.00–9.50)	4.151	0.020*
HIF-1α	8.0(7.00–12.00)	3.0(2.00–6.00)	4.151	0.000*
c-Myc	7.0(6.00–10.00)	4.0(2.00–6.50)	3.625	0.000*
PKM2	7.0(5.00–12.00)	6.0(1.50–10.00)	2.041	0.041*

Data are expressed as median (P25–P75), *P < 0.05.
