# Supplementary material for: PKM2 enhances chemosensitivity to cisplatin through interaction with the mTOR pathway in cervical cancer
Source: Sci Rep. 2016 Aug 5;6:30788. doi: 10.1038/srep30788 (PMC4974606; doi:10.1038/srep30788)
Supplement: Supplementary Information [file srep30788-s1.pdf]

Title page

Full title: PKM2 enhances chemosensitivity to cisplatin through interaction with the mTOR pathway in cervical cancer

Short title: PKM2 modulates cisplatin sensitivity in cervical cancer

Authors names: Haiyan Zhu[1], Jun Wu[1], Wenwen Zhang[1], Hui Luo[1], Zhaojun Shen[1], Huihui Cheng[1], Xueqiong Zhu[1*]

Affiliations

[1]Department of Obstetrics and Gynecology, the Second Affiliated Hospital of Wenzhou Medical University, Wenzhou 325027, China.

E-mail:

Haiyan Zhu: zhuhaiyandoc@sina.com;

Jun Wu: wujun622217@163.com;

Wenwen Zhang：zww122866@126.com;

Hui Luo：luohui19900901@163.com;

Zhaojun Shen：shenzhaojun619@163.com;

Huihui Cheng：chenghuihui0525@163.com.

*__Corresponding Authors__:

Xueqiong Zhu MD, PhD

No. 109 Xueyuan Xi Road, Department of Obstetrics and Gynecology, The Second Affiliated Hospital of Wenzhou Medical University, Wenzhou, Zhejiang, 325027, China

Tel: +86 577 88002796 (office); +86 13906640759 (mobile)

Fax: +86 577 88002560

E-mail: zjwzzxq@163.com

Supplementary figure legends

Supplementary Figure 1. Influence of PKM2 knockdown on chemosensitivity to cisplatin in C4-1 and HeLa cells. Cells were treated with different concentrations of cisplatin (0, 1.25, 2.5, 5, 10, 20μM) for 48h (A), (0, 0.31. 0.62, 1.25, 2.5, 5, 10μM) for 7 days (B). Cell viability was analyzed by MTT assay for chemosensitivity. The chemosensitivity of C4-1 and HeLa cells to cisplatin was significantly decreased by transient transfection with PKM2- siRNA-1 and PKM2- siRNA-2 compared with negative control (NC). $^*P<0.05$; $^{**}P<0.01$. (C, D) The IC_{50} value of cisplatin in siRNA- NC, PKM2- siRNA-1 or PKM2- siRNA- 2 C4-1 and HeLa cells.

Supplementary Figure 2. The interaction between PKM2 and m-TOR pathway was involved in cisplatin response. (A) After treatment with or without 40nM rapamycin and with or without 10μM cisplatin for 24h, expression of PKM2 and m-TOR in C4-1 and HeLa cells was determined by western blot analysis. (B) Western blot analysis of PKM2 and m-TOR expression in PKM2- siRNA C4-1 cells and HeLa cells with or without 10μM cisplatin treatment for 24h.

A 48hrs

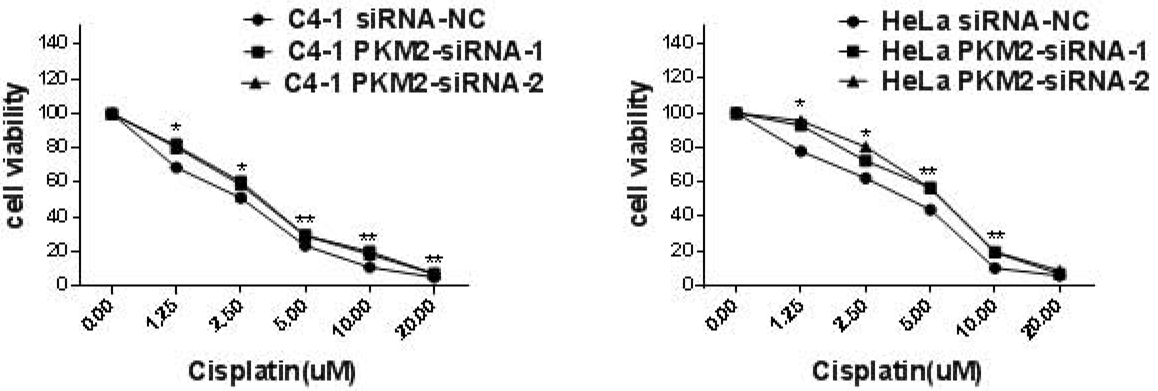

B 7days

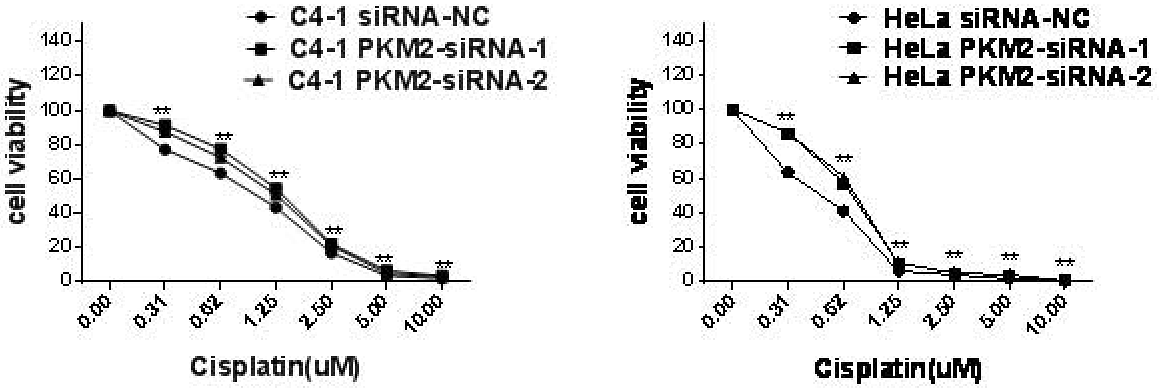

C

	siRNA-NC	PKM2-siRNA-1	PKM2-siRNA-2
48 h	2.32±0.4	3.26±0.49	3.14±0.47
5 days	1.01±0.34	1.42±0.16	1.51±0.16
7 days	0.77±0.25	1.26±0.15	1.09±0.14

D

	siRNA-NC	PKM2-siRNA-1	PKM2-siRNA-2
48 h	3.36±1.19	4.93±0.60	5.41±0.62
5 days	1.09±0.13	1.57±0.17	1.79±0.50
7 days	0.44±0.13	0.66±0.21	0.68±0.23

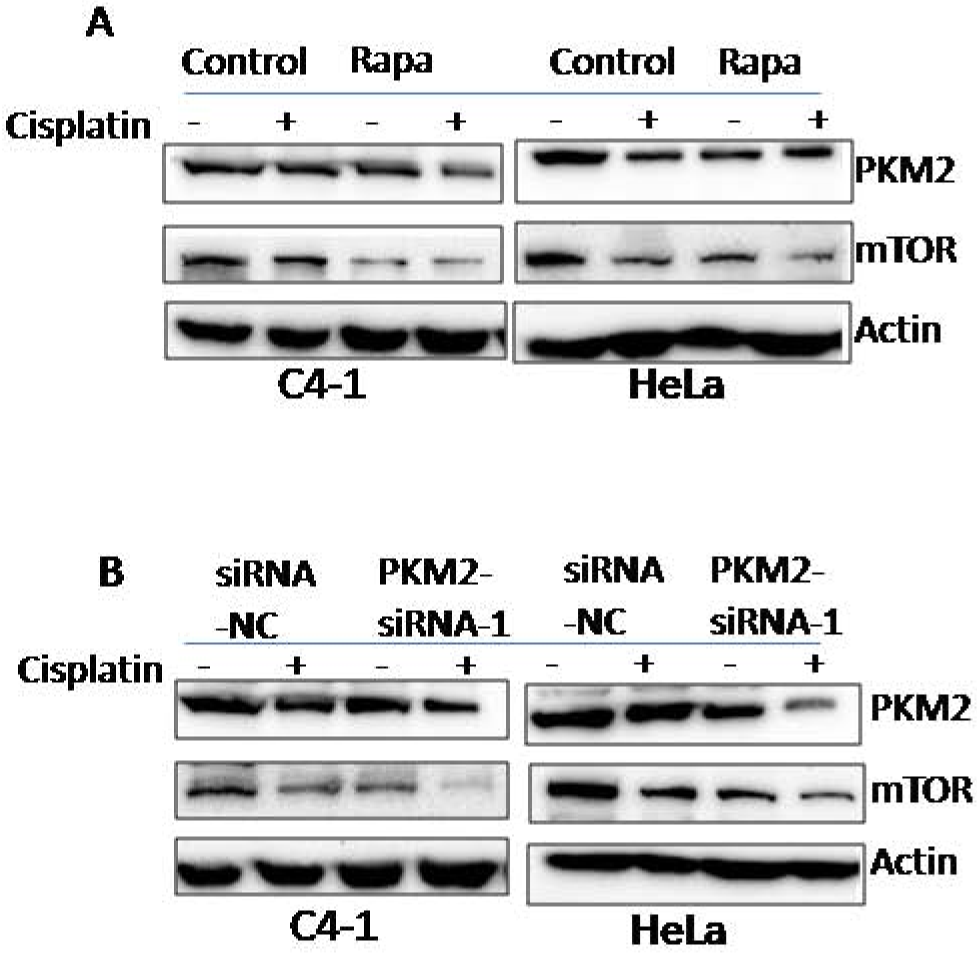
A
Control
Rapa
Control
Rapa
Cisplatin
- + - + - + - +
PKM2
mTOR
Actin
C4-1
HeLa
B
siRNA
-NC
PKM2-
siRNA-1
siRNA
-NC
PKM2-
siRNA-1
Cisplatin
- + - + - + - +
PKM2
mTOR
Actin
C4-1
HeLa
